# From planktonic to biofilm states: single-cell transcriptomics reveals metabolic reprogramming and cellular heterogeneity in *Acinetobacter baumannii*

**DOI:** 10.1016/j.bioflm.2026.100393

**Published:** 2026-08-21

**Authors:** Na Li, Yue Hu, Lulu Liu, Zhou Chen, Shuting Huang, Mingkai Li, Xinggang Mao, Xiaoyan Xue

**Affiliations:** a Department of Pharmacology, School of Pharmacy, Air Force Medical University, No.169, Changle West Road, Xi'an, Shaanxi, 710032, PR China; b Department of Neurosurgery, The First Affiliated Hospital, Air Force Medical University, No.169, Changle West Road, Xi'an, Shaanxi, 710032, PR China

**Keywords:** *Acinetobacter baumannii*, Biofilm formation, Single-cell transcriptomics, Metabolic reprogramming, Stress adaptation, Pseudotemporal trajectory

## Abstract

*Acinetobacter baumannii*, a notorious nosocomial pathogen, exhibits enhanced antibiotic resistance through biofilm formation. However, a comprehensive understanding of the heterogeneity and regulatory dynamics underlying biofilm development at single-cell resolution is lacking. This study outlines the transcriptional landscape of *A. baumannii* biofilm formation at single-cell resolution and explores potential therapeutic targets. We monitored the dynamic formation process of biofilms using single-cell RNA sequencing (scRNA-seq) technology. Subsequently, we conducted characteristic genes, gene ontology (GO) enrichment, and pseudotemporal analysis on each identified cluster. In this study, scRNA-seq and pseudotemporal trajectory results showed the transition from planktonic to biofilm states in *A. baumannii*. Increased cellular heterogeneity was observed during biofilm maturation: planktonic subpopulations (AB_0 h) displayed a metabolic divergence between phenylacetate catabolism (*paa* genes) and the tricarboxylic acid cycle (*acnD*, *atp* genes), whereas the 12 h mixed population (M_12 h) exhibited co-upregulation of ribosomal (*rpl*, *rps*) and stress response genes (*recA*, *uvrA*), facilitating protein synthesis and environmental adaptation. Mature biofilm subpopulations (BF_48 h) activated iron acquisition (*bauA*, *basD*) and sulfur/nitrogen metabolism pathways (*ssuC*, purine degradation genes) under nutrient limitation, alongside DNA repair (*uvrB*, *uvrC*) and proteostasis mechanisms (*clpB*, *clpX*). Pseudotemporal analysis identified a critical branchpoint (Node 2) that marked the transition from the high-metabolism planktonic to the low-metabolism biofilm state, characterized by the downregulation of ribosomal (*rpl*, *rps*) and transporter (*putP*) genes. These findings characterize transcriptional programs associated with biofilm maturation and reveal subpopulation-specific metabolic features that may represent potential vulnerabilities warranting further investigation through targeted mutagenesis and functional assays.

## Highlights

• First scRNA-seq atlas of *A. baumannii* biofilm reveals metabolic heterogeneity at single-cell resolution.
• Metabolic plasticity in planktonic cells: coexistence of phenylacetate and TCA cycle subpopulations.
• Early biofilm formation couples ribosomal upregulation with stress response gene expression.
• Mature biofilm activates sulfur/iron scavenging, DNA repair, and proteostasis to sustain stability.
• Pseudotemporal analysis identifies a bifurcation point and key regulators (*putP*, ribosomal genes).

## 1 Introduction

Infections occurring 48–72 h after patient admission are defined as nosocomial infections, which are associated with increased morbidity and mortality [1]*.* Among nosocomial pathogens, *Acinetobacter baumannii* has emerged as a major global threat to healthcare [2]. This ubiquitous Gram-negative opportunist causes ventilator-associated pneumonia, central venous catheter-related bloodstream infections, skin and soft tissue infections, surgical site infections, and catheter-associated urinary tract infections [3]. It is associated with high mortality and rapidly develops multidrug resistance. The World Health Organization (WHO) listed *A. baumannii* as a critical-priority pathogen in 2017 and reaffirmed this status in 2024 [4].

A major contributor to its persistence and spread is biofilm formation, which plays a central role in the pathogenesis of nosocomial and recurrent infections. Biofilms are structured microbial communities encased in extracellular polymeric substances (EPS) [5]. This mode of growth markedly reduces bacterial susceptibility to antibiotics and host immune defenses, owing to low metabolic activity and the physical barrier provided by the EPS matrix [6]. The ability to form biofilms allows *A. baumannii* to colonize host tissues, persist on medical devices, and survive in harsh environments, thereby complicating therapeutic interventions [7]. Biofilm-associated infections, particularly ventilator-associated pneumonia and catheter-related infections, display extreme antibiotic resistance and present major challenges to clinical management [8].

Biofilm development typically progresses through initial attachment to biotic or abiotic surfaces, microcolony formation, maturation, and detachment, and is routinely evaluated by qualitative assays (tube, tissue-culture plate) or quantitative assays (crystal violet staining), complemented by scanning electron microscopy (SEM) visualization [8]. In addition to these well-established phenotypic assessments, most studies of *A. baumannii* biofilms rely on population-level “bulk” omics approaches or targeted investigations of specific genes. These methods generally assume cellular homogeneity and therefore obscure transcriptional heterogeneity within the biofilm [9]. In reality, biofilms are highly structured multicellular communities whose development and stability likely depend on functional specialization among distinct subpopulations [10]. However, this cellular heterogeneity remains unexplored at single-cell resolution in *A. baumannii*. Moreover, conventional antibiotics exhibit limited efficacy against established biofilms [11], which show broad tolerance to β-lactams, quinolones, aminoglycosides, and macrolides [12]. Biofilm formation is a multifactorial process regulated by numerous determinants, including adhesins, capsular polysaccharides, surface appendages, biofilm-associated protein (Bap), outer membrane protein A (OmpA), chaperone-usher pili, iron uptake mechanism, poly-β-(1,6)-N-acetylglucosamine (PNAG), two-component system, quorum-sensing system, and extrinsic factors [5,13]. Although promising approaches such as quorum-sensing inhibitors [14], antimicrobial peptides [15], phage therapy [14] and photodynamic therapy [16] are under investigation, issues with biocompatibility, bioavailability, and specificity limit their clinical translation [14]. A deeper, more nuanced understanding of biofilm architecture and functional heterogeneity is therefore critical to guide the development of effective therapeutic strategies.

To address these limitations, we applied single-cell RNA sequencing (scRNA-seq) to profile the transcriptional landscape of *A. baumannii* biofilm development at the individual-cell level, capturing cellular heterogeneity and dynamic changes in gene expression over time. Pioneering studies, such as investigations of *Escherichia coli* persister cells [17] and *A. baumannii*-induced ferroptosis in macrophages [18], have demonstrated the value of scRNA-seq in uncovering novel microbial behaviors. In this study, we generated the first scRNA-seq atlas of *A. baumannii* biofilm development and revealed shifts in cell subpopulations and differential gene expression across developmental stages. Pseudotime trajectory analysis identified key transition points from the planktonic (free-living) to the biofilm state. This study provides unprecedented insights into biofilm heterogeneity and identifies potential targets for antibiofilm therapies.

## 2 Methods

### 2.1 Bacterial culture

*A. baumannii* (ATCC 19606) was obtained from Bena Culture Collection Ltd. Co. (Beijing, China). *A. baumannii* was cultured in Luria–Bertani (LB) medium at 37 °C with shaking at 220 rpm until reaching the logarithmic growth phase. The bacterial suspension was adjusted to 5 × 10^6^ CFU/mL for downstream experiments.

### 2.2 Crystal violet staining for biofilm biomass quantification

Bacterial suspension was inoculated into 24-well plates (1 mL/well) and incubated statically at 37 °C for 2, 4, 12, 24, 48, 72, and 96 h. After incubation, the supernatant was removed and each well was gently washed with 1 mL of sterile phosphate-buffered saline (PBS; pH 7.4). Biofilms were stained with 1 mL of 0.1% crystal violet for 15 min, then rinsed with PBS three times and air-dried. The bound dye was dissolved in 1 mL of 95% ethanol per well, and absorbance was measured at 630 nm using a microplate reader (BioTek, USA) [19].

### 2.3 Fluorescence microscopy

Bacterial suspension was inoculated into 24-well plates containing sterile round coverslips (1 mL/well). After static incubation at 37 °C for 4, 12, 48, and 96 h, 10 μL of fluorescein isothiocyanate (FITC; 10 mg/mL) was added to each well. Following gentle mixing, plates were incubated for an additional 2 h at 37 °C [20]. Coverslips were then removed, rinsed three times with sterile PBS, and mounted on slides with the biofilm-facing side down for observation under a fluorescence microscope(Zeiss, Germany).

### 2.4 SEM

Bacterial suspension was inoculated into 24-well plates containing sterile round coverslips (1 mL/well). After static incubation at 37 °C for 12 and 48 h, the supernatant was discarded and each well was gently rinsed with 1 mL of sterile PBS three times, followed by fixation with 1 mL of 3% glutaraldehyde at 4 °C for 12 h. Concurrently, planktonic bacteria at logarithmic growth phase and at 12 h were collected by centrifugation (1990 × g, 5 min), and the pellets were resuspended in fixative and stored at 4 °C for 12 h. An appropriate volume of the fixed suspension was placed onto coverslips and air-dried. All samples were then processed for SEM (Thermo Fisher Scientific, Czech Republic) observation.

### 2.5 Confocal laser scanning microscope (CLSM)

Bacterial suspension was inoculated into 24-well plates containing sterile round coverslips (1 mL/well) and incubated statically at 37 °C for 48 h. After biofilm formation, the supernatant was discarded and the biofilms were gently rinsed with 1 mL of sterile PBS. Then, 200 μL of FITC-conjugated concanavalin A (FITC-conA; 50 μg/mL) was added to each well, and plates were incubated at 37 °C for 3 h. After aspiration, 200 μL of Hoechst 33342 (20 μg/mL) was added and incubated at room temperature for 15 min. Coverslips were rinsed with sterile PBS and air-dried, then mounted on slides with 4 μL of anti-fade mounting medium. Samples were observed under CLSM with excitation/emission at 488 nm/520–530 nm for FITC and 350 nm/461 nm for Hoechst 33342. Z-stack images were acquired at 1 μm intervals, and 3D reconstruction was performed using the microscope software.

### 2.6 ScRNA-seq: library preparation and analysis

Sequencing and bioinformatic analyses were performed by Majorbio Co., Ltd. (Shanghai, China). To evaluate the reproducibility of subpopulation structures across independent experiments, two biological replicates were processed for the 48 h time point (Pre_BF_48 h and BF_48 h), while one sample per group was sequenced for the remaining time points (AB_0 h and M_12 h). Details of sample preparation, library construction, sequencing, and primary data analysis are provided in **Supplementary Data 1**.

### 2.7 **Real-time quantitative reverse transcription polymerase chain reaction analysis** (RT-qPCR analysis)

Bacteria were pelleted (1990 g, 5 min), and total RNA was extracted with TRIzol reagent (APExBIO, USA). Complementary DNA (cDNA) was synthesized using PrimeScript RT Master Mix (APExBIO, USA). Quantitative PCR was performed on an Agilent system (USA) using SYBR Green PCR Master Mix (APExBIO, USA), with *16S rRNA* as the reference gene. Relative mRNA expression was calculated with the 2^−ΔΔCt^ method [21]. Primers (Sangon Biotech, Shanghai, China) are listed in Table S1 (Supplementary Data 2).

### 2.8 Statistical analysis

Statistical analyses were performed using GraphPad Prism 10.1 (GraphPad Software, USA). Normality and homogeneity of variances were evaluated using the Shapiro–Wilk and Brown–Forsythe tests, respectively. For single-cell expression data that were non-normally distributed, differences among the three clusters were assessed on a per-gene basis using the Kruskal–Wallis test, followed by false-discovery-rate (FDR) correction. Genes with FDR < 0.05 were further analyzed with Dunn's post-hoc test, with Bonferroni adjustment for pairwise comparisons. For normally distributed data, biofilm fluorescence intensity across different time points and relative expression levels of genes among the three samples were both analyzed using one-way ANOVA with Tukey's post-hoc test. Statistical significance was set at *P* < 0.05. Error bars represent standard deviation (SD) of biological replicates. All experiments were performed in biological triplicates, unless otherwise stated. *P < 0.05, **P < 0.01, ***P < 0.001, ****P < 0.0001, ns indicates no significant difference.

## 3 Results

### 3.1 Dynamic process of *A. baumannii* biofilm formation

**Fig. 1:**
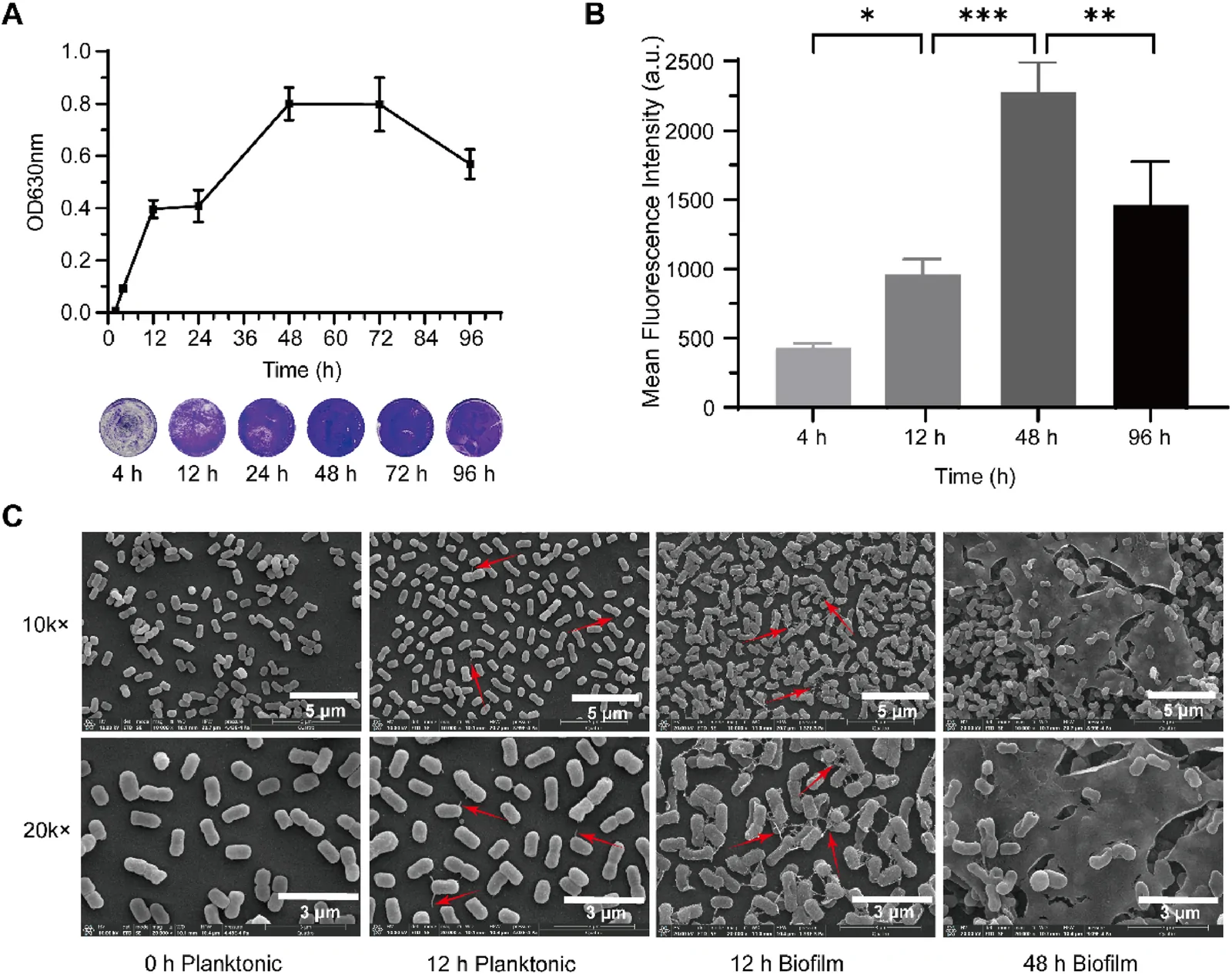
Dynamic monitoring of *A. baumannii* ATCC 19606 biofilm formation. A) Crystal violet staining to quantify biofilm biomass at indicated time points. B) Quantification of FITC fluorescence intensity of biofilm at 4, 12, 48, and 96 h. C) SEM images of planktonic (0 h), planktonic (12 h), biofilm (12 h), and mature biofilm (48 h) cells. Scale bars: 5 μm (10k×); 3 μm (20k×). Red arrows highlight roughened surfaces and filamentous intercellular connections of the bacteria. (For interpretation of the references to color in this figure legend, the reader is referred to the Web version of this article.)

Biofilm biomass of *A. baumannii* ATCC 19606 was quantified using crystal violet staining. Initial attachment was detected at 4 h, with thin films visible at 12 h. Biomass accumulation peaked at 48 h (plateau phase) and declined after 72 h (Fig. 1A). FITC fluorescence labeling of biofilms on glass slides corroborated these dynamics, showing maximal intensity at 48 h and a subsequent reduction at 96 h (Fig. 1B). SEM further revealed morphological transitions: planktonic cells (0 h) exhibited smooth, blunt-ended surfaces, 12 h samples displayed roughened surfaces with filamentous intercellular connections, and mature biofilms (48 h) were fully embedded in EPS layers (Fig. 1C). CLSM observation of the 48 h biofilms revealed that bacterial cells were tightly packed within multilayered, matrix-encased communities, confirming that 48 h culture system supports the formation of structurally mature biofilms (Fig. S1, Supplementary Data 2). Together, these findings demonstrate the dynamic course of *A. baumannii* biofilm development, which underpins a deeper mechanistic understanding.

### 3.2 ScRNA-seq reveals enhanced heterogeneity in *A. baumannii* biofilms

**Fig. 2:**
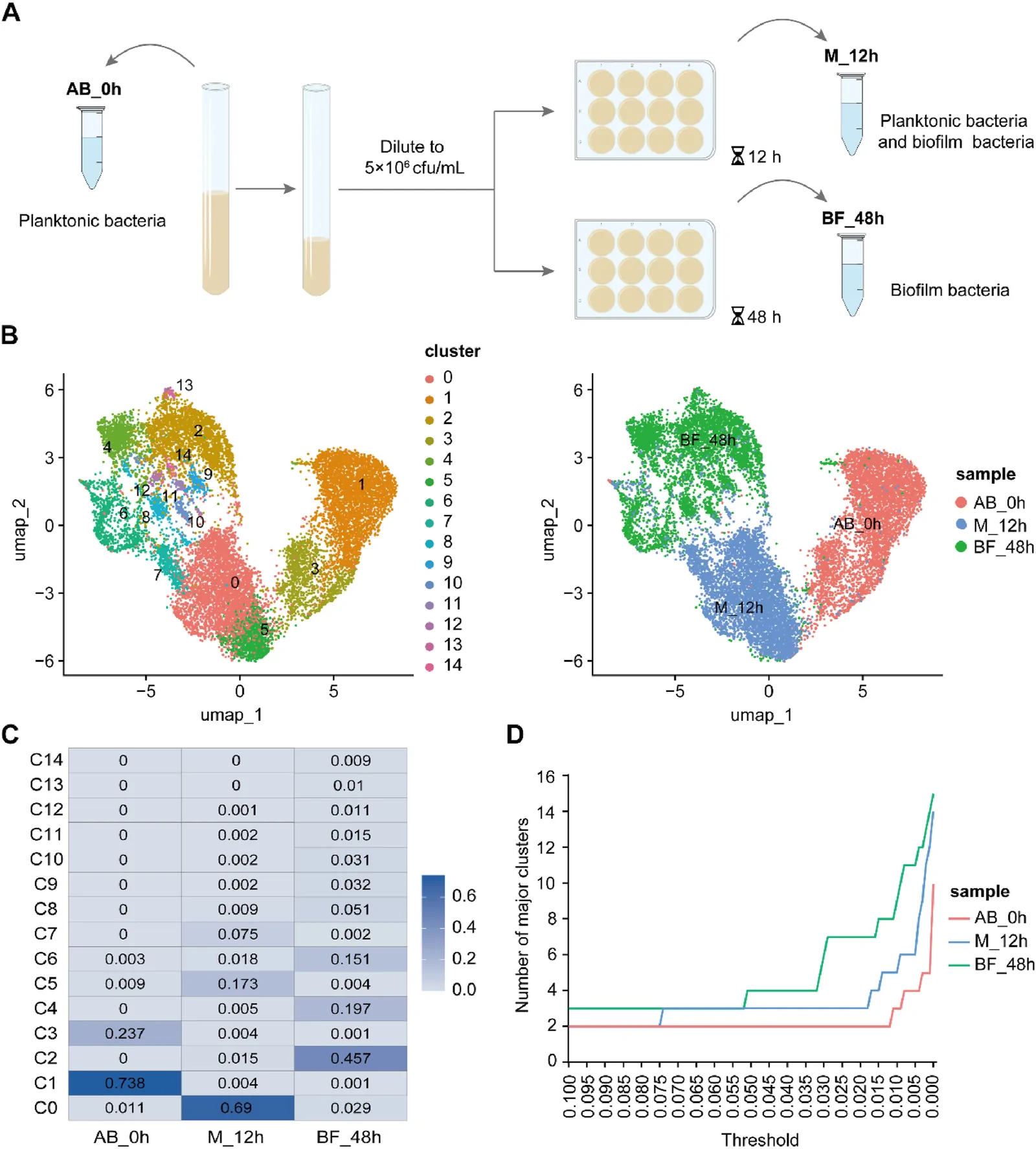
Cellular heterogeneity revealed by scRNA-seq. A) Schematic of scRNA-seq sampling. B) UMAP visualization of bacterial subpopulations (colors represent different clusters and samples). C) Heatmap of cluster-wise relative abundance across samples (darker shades indicate higher proportions). D) Subpopulation counts under different abundance thresholds. (For interpretation of the references to color in this figure legend, the reader is referred to the Web version of this article.)

To investigate the molecular basis of *A. baumannii*'s transition from planktonic to biofilm states, three populations—planktonic cells (AB_0 h), transitioning cells (M_12 h, a mixture of planktonic and early biofilm bacteria), and mature biofilm cells (BF_48 h)—were analyzed using scRNA-seq with random primer-based whole-transcriptome amplification (Fig. 2A). Following quality control, the median unique molecular identifier (UMI) counts per cell were 229 (AB_0 h), 178 (M_12 h), and 68 (BF_48 h), with corresponding median gene counts of 158, 127, and 44 (Table S2, Supplementary Data 2). After removing dead cells, empty droplets, and doublets, 14,600 high-quality cells were retained. The marked reduction in transcriptional activity in BF_48 h is consistent with metabolic dormancy associated with mature biofilms. Despite inherent limitations of microbial rRNA in mRNA capture, sequencing saturation exceeded 40% (Table S2, Supplementary Data 2), supporting reliable representation of biological states, consistent with prior work [22]. To assess the reproducibility between batches, we compared the BF_48 h dataset with the pre-experiment dataset (Pre_BF_48 h) that was cultivated under the same conditions. The two datasets showed consistent uniform manifold approximation and projection (UMAP) coordinates, similar clustering partitions, and strong correspondence between matched subclusters (Fig. S2, Supplementary Data 2), confirming that major subpopulations are robustly conserved across biological replicates.

UMAP identified 15 transcriptionally distinct clusters across three samples (Fig. 2B). AB_0 h cells were predominantly clustered into two subpopulations (Cluster 1: 73.8%; Cluster 3: 23.7%), whereas M_12 h exhibited increased diversity, with three notable clusters (Cluster 0: 69.0%; Cluster 5: 17.3%; Cluster 7: 7.5%). The BF_48 h biofilm displayed maximal heterogeneity, with nine clusters exceeding 1% abundance. Biofilm-associated clusters (e.g., Cluster 2: 45.7%; Cluster 4: 19.7%; Cluster 6: 15.1%) were largely distinct from planktonic-dominant clusters, indicating fundamentally different transcriptional programs (Fig. 2C).

To quantify this diversity, we analyzed the number of detectable subpopulations across a range of abundance thresholds. BF_48 h consistently exhibited greater diversity than M_12 h and AB_0 h, confirming a progressive increase in transcriptional heterogeneity during biofilm development (Fig. 2D). These findings indicate that biofilm formation is not a uniform process but entails diversification into transcriptionally specialized subpopulations.

### 3.3 Metabolic heterogeneity in planktonic subpopulations enhances adaptability

**Fig. 3:**
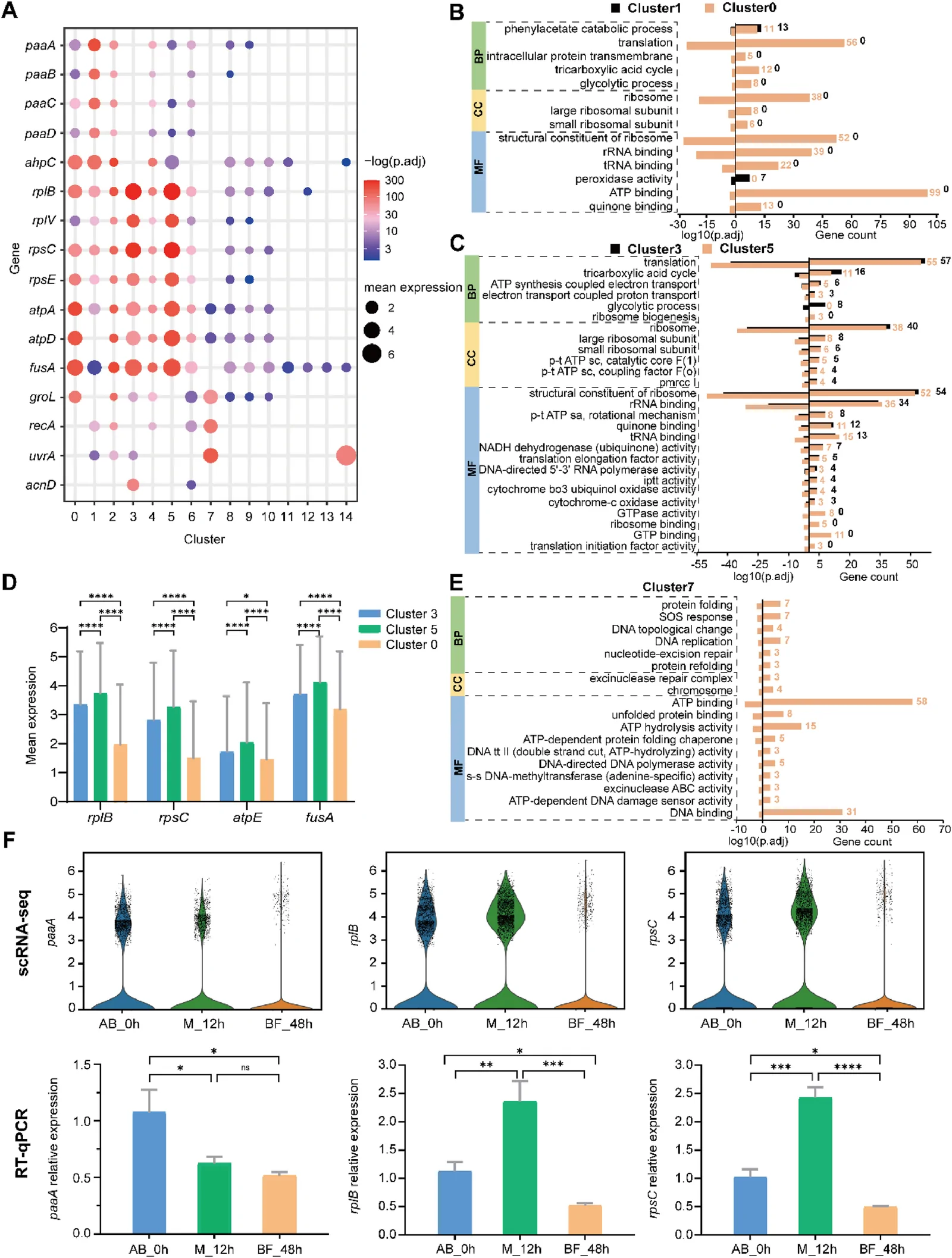
Functional characterization of AB_0 h and M_12 h subpopulations. A) Bubble plot of differentially expressed genes (DEGs). circle size = expression level; color intensity = statistical significance. B) GO enrichment of Cluster 1 and 0 (BP: Biological Process; MF: Molecular Function; CC: Cellular Component). C) GO enrichment of Cluster 3 and 5 (p-t ATP sc: proton-transporting ATP synthase complex; pmrcc I: plasma membrane respiratory chain complex I; p-t ATP sa: proton-transporting ATP synthase activity; iptt activity: inorganic phosphate transmembrane transporter activity). D) Mean expression of *rplB*, *rpsC*, *atpE*, and *fusA* in Clusters 3, 5, and 0. E) GO enrichment of Cluster 7 (DNA tt II activity: DNA topoisomerase type II activity; s-s DNA-methyltransferase activity: site-specific DNA-methyltransferase activity). F) ScRNA-seq violin plots (top) of *paaA*, *rplB*, and *rpsC* expression across the three samples and bar graphs of RT-qPCR validation (bottom). In violin plots, Y-axis represents gene expression level; Each violin = sample; black dots = individual cells; width = kernel density distribution. (For interpretation of the references to color in this figure legend, the reader is referred to the Web version of this article.)

Marker genes and GO enrichment analyses of AB_0 h clusters revealed functional specialization. Cluster 1 (73.8% of AB_0 h) showed enrichment of genes for phenylacetate catabolic process (*paaA*, encoding phenylacetyl-CoA epoxidase; *paaB*, encoding phenylacetyl-CoA ligase) and peroxidase activity (*ahpC*, encoding alkyl hydroperoxide reductase subunit C) (Fig. 3A and B). The *paa* gene cluster enables phenylacetate oxidation to acetyl-CoA and succinyl-CoA, sustaining carbon and energy acquisition under nutrient stress [23]. Disruption of this pathway weakens stress adaptation, reducing antibiotic resistance and hydrogen peroxide tolerance [24]. AhpC detoxifies organic peroxides by reducing alkyl hydroperoxides to alcohols, highlighting its role in antioxidation. Its upregulation suggests elevated reactive oxygen species (ROS) in this subpopulation, likely generated during aerobic metabolism, necessitating strong detoxification defenses.

Cluster 3 (23.7% of AB_0h) displayed a distinct profile, with elevated ribosomal genes (*rpl*, encoding the large subunit of the ribosome; *rps,* encoding the small subunit of the ribosome) and genes associated with energy production, including *atpB*/*atpD* (encoding adenosine triphosphate (ATP) synthase subunits) and *acnD* (encoding iron/sulfur-dependent 2-methylisocitrate dehydratase) (Fig. 3A). GO enrichment analysis further supported that Cluster 3 was highly associated with protein synthesis (structural constituent of ribosome, translation), tricarboxylic acid (TCA) cycle, glycolytic process, and aerobic respiration (quinone binding, cytochrome-c oxidase activity) (Fig. 3C). These features indicate a metabolically active subpopulation primed for rapid growth and protein synthesis.

Together, these data reveal functional specialization within the seemingly homogeneous planktonic population. The major subpopulation (Cluster 1) specializes in stress resilience through alternative carbon metabolism and antioxidation, ensuring survival under adverse conditions. The minor subpopulation (Cluster 3) emphasizes rapid proliferation through biosynthesis and energy production. This metabolic dichotomy equips *A. baumannii* with plasticity to balance survival and growth depending on environmental conditions. The coexistence of these subpopulations implies an ecological trade-off between energy allocated for maintenance versus biomass expansion. The regulatory mechanisms and potential interactions, such as metabolic cross-feeding, remain unclear. Future integration of spatial metabolomics and single-cell proteomics is required to assess whether metabolic intermediates (e.g., acetyl-CoA) are shared across subpopulations to coordinate phenotypic plasticity. Such insights could inform strategies to disrupt metabolic synergy in *A. baumannii* biofilms, a critical avenue in combating multidrug-resistant infections.

### 3.4 Enhanced protein synthesis and stress response activation during the transition from planktonic to biofilm state

ScRNA-seq profiling of the transitional M_12 h population revealed functional diversification into distinct subpopulations. The two predominant clusters, Cluster 0 (69.0% of M_12 h) and Cluster 5 (17.3% of M_12 h), both displayed upregulation of ribosomal (*rpl*, *rps*) and ATP-related (*atp*) genes, indicating heightened biosynthetic and energetic activity (Fig. 3A). Notably, mean expression levels of these genes in Cluster 5 exceeded those in Cluster 3 (AB_0 h) and Cluster 0 (M_12 h) (Fig. 3D). Cluster 5 was uniquely characterized by high expression of *fusA* (Fig. 3A and D), encoding elongation factor EF-Tu, a guanosine triphosphatase (GTPase) essential for delivering aminoacyl-tRNAs to the ribosome during translational elongation [25]. Accordingly, GO terms for translation elongation factor activity, GTPase activity, and GTP binding were specifically enriched in Cluster 5 (Fig. 3C). This transcriptional profile suggests that Cluster 5 represents a hyper-proliferative state, with an exceptionally high demand for protein synthesis to sustain rapid bacterial replication. Although also enriched in translation-related functions, Cluster 0 uniquely retained the phenylacetate catabolic process (e.g., *paa* genes) characteristic of the planktonic Cluster 1 (Fig. 3A and B). This persistence suggests that alternative carbon metabolism remains important during early biofilm development, consistent with prior studies in *Acinetobacter* strains [24].

In contrast, a smaller but critical subpopulation, Cluster 7 (7.5% of M_12 h), was specialized for stress mitigation. This cluster exhibited strong upregulation of genes encoding the molecular chaperone GroEL (*groL*) and DNA repair enzymes (*recA*, *uvrA*) (Fig. 3A)**.** This coordinated program indicates a dual protective strategy: proteostasis maintenance via GroEL-mediated refolding and genotoxic stress repair through RecA-mediated Save Our Soul response (SOS response) and UvrA-dependent nucleotide excision repair (NER) (Fig. 3E). The NER system is evolutionarily conserved for excision of diverse DNA lesions [26]. Induction of *recA* is particularly significant, as it orchestrates error-prone DNA repair and promotes SOS-driven phenotypic diversification, thereby priming the population for adaptation [27]. The essential role of molecular chaperones in biofilm integrity is supported by findings that *groL* deficiency disrupts biofilm formation in *Cronobacter sakazakii* [28]. Functional annotation positioned Cluster 7 at the intersection of energy expenditure and stress management, with enrichment for ATP-dependent processes including protein folding, DNA damage sensing, and SOS response (Fig. 3E). We propose that Cluster 7 represents a metabolically active, homeostasis-focused subpopulation, in which ATP hydrolysis fuels protein and DNA quality-control systems, thereby sustaining early biofilm establishment under stress.

Collectively, these findings suggest that the planktonic-to-biofilm transition is orchestrated through a metabolic–stress trade-off. The bacterial population expands proliferative subpopulations (Clusters 0 and 5) to drive growth and matrix production, while dedicating a smaller fraction (Cluster 7) to stress adaptation, ensuring survival under fluctuating conditions.

To validate the robustness of the scRNA-seq results, RT-qPCR was performed for differentially expressed genes (*paa*, *rpl*, *rps*). Expression patterns across AB_0 h, M_12 h, and BF_48 h were highly consistent with sequencing data (Fig. 3F). *paaA* expression peaked in the AB_0 h phase, reinforcing the metabolic heterogeneity intrinsic to planktonic populations. Conversely, *rpl* and *rps* exhibited maximal expression in the transitional M_12 h phase, followed by reduced activity in mature biofilms. This expression gradient validates the increased translational demand during biofilm initiation and the subsequent metabolic shift from growth to maintenance in mature biofilm.

### 3.5 Biofilm-specific survival strategies: sulfur/nitrogen/iron metabolism, DNA repair and proteostasis

ScRNA-seq analysis of mature biofilm (BF_48 h) revealed a complex ecosystem of functionally specialized subpopulations, each employing distinct strategies to sustain community resilience.

**Fig. 4:**
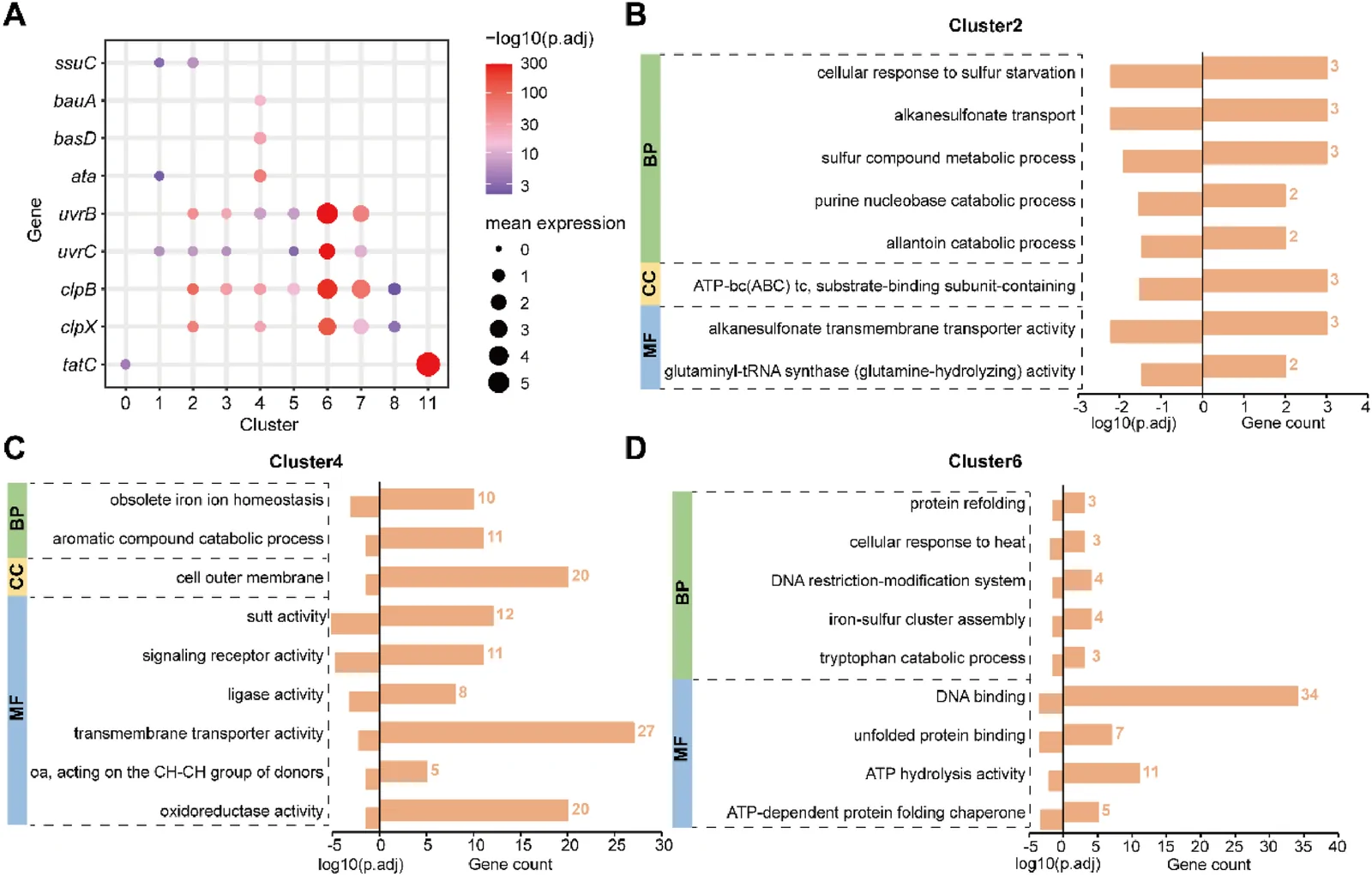
Functional characterization of BF_48 h subpopulations. A) Bubble plot of DEGs. B) GO enrichment of Cluster 2 (ATP-bc (ABC) tc: ATP-binding cassette (ABC) transporter complex). C) GO enrichment of Cluster 4 (sutt activity: siderophore uptake transmembrane transporter activity; oa: oxidoreductase activity). D) GO enrichment of Cluster 6.

Cluster 2, the predominant subpopulation (45.7% of BF_48 h), was defined by strong activation of *ssuC* (encoding alkylsulfonate permease), a key gene in sulfur salvage under nutrient limitation [29] (Fig. 4A). GO enrichment confirmed functions associated with sulfur starvation, alkanesulfonate transport, and sulfur compound metabolism (Fig. 4B). Concurrently, this cluster upregulated purine nucleobase and allantoin catabolism process (Fig. 4B), a nitrogen-scavenging pathway crucial for microbial nitrogen acquisition. In pathogens such as *Klebsiella pneumoniae*, allantoin metabolism enhances pathogenicity and antimicrobial resistance [30,31]. The co-activation of sulfur and nitrogen salvage pathways highlights a sophisticated metabolic adaptation that supports persistence under nutrient scarcity.

Cluster 4 (19.7% of BF_48 h) exhibited a distinct iron-acquisition phenotype. It showed upregulation of *bauA* (encoding the ferric-acinetobactin receptor) and *basD* (encoding the acinetobactin biosynthesis enzyme) (Fig. 4A). BauA is essential for high-affinity iron uptake under restriction, and its disruption severely impairs growth [32]. Overexpression of *bauA* and *basD* enhances biofilm maturation and resistance in *A. baumannii* [33,34]. GO enrichment highlighted siderophore uptake transmembrane transporter activity and obsolete iron ion homeostasis (Fig. 4C). Notably, co-upregulation of *ata* (encoding autotransporter) (Fig. 4A), a mediator of biofilm adhesion, immune evasion, and extracellular matrix interaction [35], suggests a coordinated iron-scavenging and virulence program driving biofilm resilience under host-imposed iron limitation.

Cluster 6 (15.1% of BF_48 h) was characterized by stress adaptation, with upregulation of NER genes (*uvrB*, *uvrC*), which excise DNA lesions [36], and ATP-dependent chaperones (*clpB*, *clpX*) for protein disaggregation and turnover (Fig. 4A). GO analysis showed enrichment for DNA repair, ATP-dependent chaperone activity, and response to heat stress (Fig. 4D). This suggests a dedicated role in maintaining genomic integrity and proteostasis under duress. Functional overlap with transitional Cluster 7 (M_12 h) implies either lineage continuity or convergent adaptation for long-term stress management.

Minor subpopulations further exemplified diversification:

1) Cluster 8 (5.1% of BF_48 h) shared the sulfur starvation response of Cluster 2 but uniquely activated pathways for mercury detoxification and cellular redox homeostasis, enabling survival under heavy metal and oxidative stress (Fig. S3A, Supplementary Data 2).
2) Cluster 10 (3.1% of BF_48 h) exhibited a sophisticated metabolic network characterized by enrichment of amide biosynthetic process, secondary metabolite biosynthetic process, and organonitrogen compound biosynthetic process (Fig. S3B, Supplementary Data 2), suggesting metabolic plasticity that supports adaptation and antimicrobial competition.
3) Cluster 11 (1.5% of BF_48 h) exhibited a significant upregulation of *tatC* (encoding the twin-arginine translocation system component) (Fig. 4A), driving the Tat-mediated transport of folded proteins across membranes (Fig. S3B, Supplementary Data 2), a process which is essential for biofilm stability. In *Pseudomonas aeruginosa*, Tat system disruption reduces extracellular DNA (eDNA) release, quorum-sensing signaling (PQS), and aminoglycoside tolerance [37,38], positioning Tat as a key determinant of biofilm stability and drug evasion.
4) Cluster 12 (1.1% of BF_48 h) displayed a functionally distinct signature centered on cell envelope biogenesis and redox metabolism. This subpopulation was enriched in Kdo2-lipid A biosynthetic process, 3-oxoacyl-[acyl-carrier-protein] synthase activity, phosphopantetheine binding, flavin reductase (NADH) activity, and identical protein binding (Fig. S4, Supplementary Data 2). This coordinated enrichment strongly suggests that Cluster 12 is actively engaged in outer membrane remodeling and energetic adjustment, potentially representing a specialized state poised for envelope stress resilience or persistence within the mature biofilm.
5) Cluster 13 (1.0% of BF_48 h), though equally rare, adopted a markedly different strategy, with significant enrichment solely for porin activity (Fig. S4, Supplementary Data 2). Modulation of porin expression directly impacts outer membrane permeability, thereby influencing the flux of nutrients, metabolic waste, and antimicrobial agents [39]. In biofilms, modulation of porin abundance may serve as a regulatory mechanism that restricts antibiotic influx while maintaining essential solute exchange, thereby contributing to the collective drug tolerance of the community.

At biofilm maturity, bacteria deploy multifaceted survival programs to counter nutrient deprivation. Iron acquisition via siderophore (e.g., acinetobactin in *A. baumannii*) is central, supporting adhesion, exopolysaccharide production, and microbial symbiosis [40]. Concurrent sulfur and nitrogen metabolism, exemplified by thiosulfate-enhanced anaerobic respiration and biofilm protein abundance, shapes biofilm structure [41]. Thiosulfate's role as both electron donor in denitrification and modulator of protein content highlights the metabolic interdependencies underpinning biofilm functionality, though the precise mechanisms remain to be elucidated.

### 3.6 Pseudotime trajectory reconstruction revealed the biofilm maturation dynamics

**Fig. 5:**
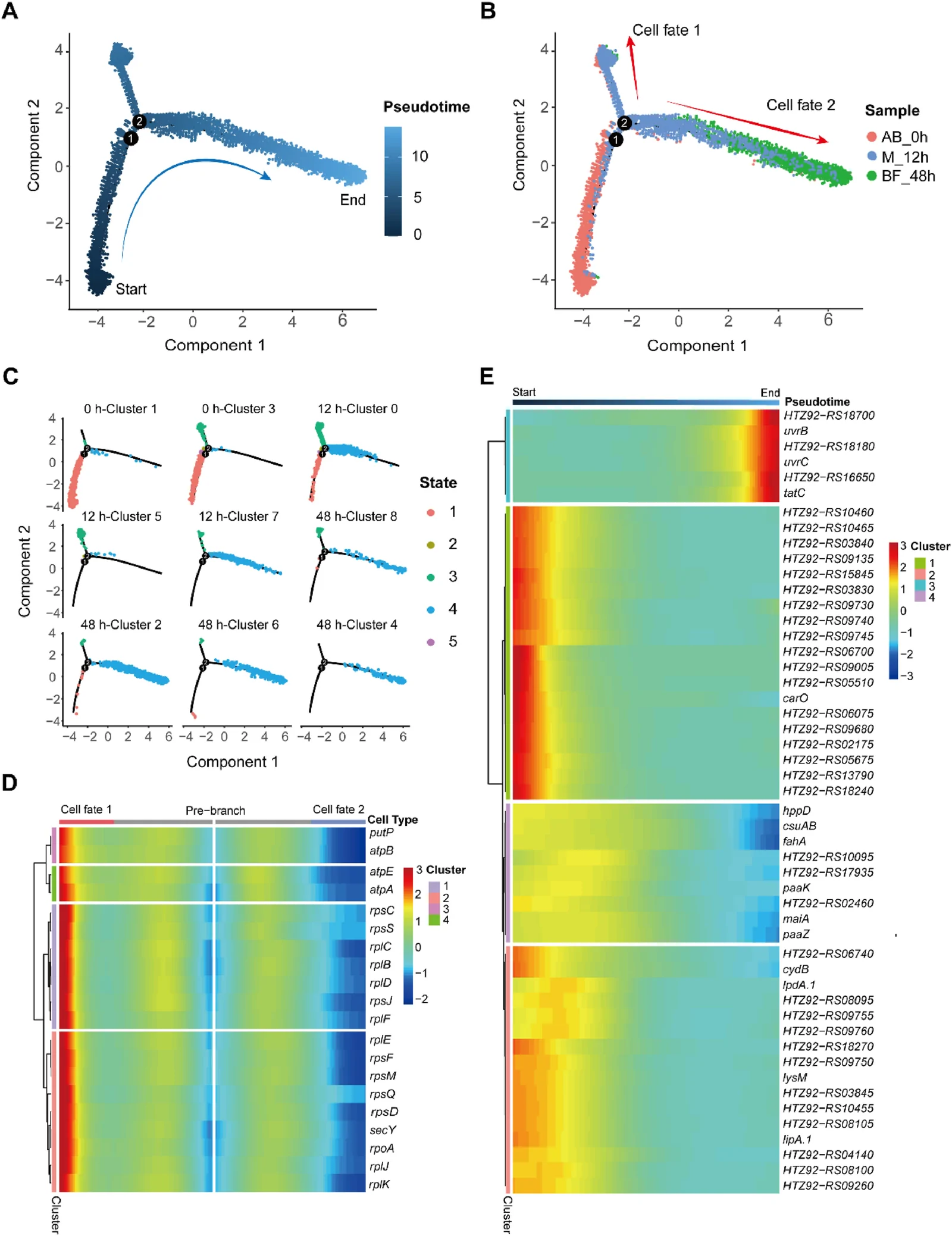
Pseudotime trajectory analysis of planktonic-to-biofilm transition in *A. baumannii.* A) Pseudotime trajectories predicted by Monocle2. B) Mapping of bacterial trajectories across samples. C) Mapping of trajectories to selected clusters. D) Branch point 2 analysis: heatmap of DEGs defining fate decisions. The middle panel represents the pre-branch state, with the left branch corresponding to Fate 1 and the right branch to Fate 2. E) Heatmap of DEGs across pseudotime, with pseudotime on the horizontal axis, genes on the vertical axis, and expression levels indicated by color gradients (red = high, blue = low). (For interpretation of the references to color in this figure legend, the reader is referred to the Web version of this article.)

To model transcriptional transitions during biofilm development, we reconstructed a pseudotime trajectory. Cells near the origin (darker hues) represented early stages, whereas distal cells (lighter hues) corresponded to mature states (Fig. 5A). The sequential ordering of AB_0 h, M_12 h, and BF_48 h samples along this trajectory recapitulated the chronological progression of biofilm assembly (Fig. 5B), validating the trajectory model.

A critical bifurcation was identified at Node 2, where cells diverged into two branches: one aligned with planktonic maintenance (Cell fate 1), the other with biofilm maturation (Cell fate 2) (Fig. 5B). Transitional clusters such as M_12 h Cluster 0 bifurcated here, whereas mature biofilm clusters (BF_48 h) and the stress-adapted Cluster 7 committed exclusively to Fate 2 (Fig. 5C). Transcriptional programs distinguished the two fates. Fate 1 retained high metabolic activity, upregulating ribosomal (*rpl*, *rps*) and transporter (*putP*) genes. In contrast, Fate 2 suppressed these processes, reflecting a shift to metabolic quiescence favoring matrix integrity over growth (Fig. 5D).

Analysis of pseudotime-dependent gene expression reinforced this metabolic partitioning. Genes progressively upregulated along pseudotime (e.g., *uvrB*, *uvrC*, *tatC*) were enriched for stress resilience and matrix maintenance (Fig. 5E). Conversely, genes associated with aromatic compound catabolism (e.g., *paaK*, *paaZ*), early adhesion factors (e.g., *csuAB*), and lipid metabolism (e.g., *lpdA*) were coordinately downregulated (Fig. 5E). This shift marks a wholesale reprogramming of metabolism and structure during maturation. Notably, several dynamically expressed genes remain uncharacterized, yet their co-expression with biofilm regulators such as *tatC* suggests novel roles in biofilm development that merit future functional investigation.

## 4 Discussions and conclusions

This study pioneered the systematic dissection of *A. baumannii* biofilm ontogeny through single-cell transcriptomics, unraveling a dynamic regulatory network driven by metabolic reprogramming, stress adaptation, and cellular heterogeneity. The key findings of this study reveal that planktonic bacteria employ a metabolic division of labor between phenylacetate catabolism and TCA cycle-dependent coexisting subpopulations, which enhances their environmental adaptability. During biofilm initiation, ribosomal (*rpl/rps*), and stress-responsive (*groL/recA*) genes are co-expressed, achieving a balance between rapid proliferation and environmental stress tolerance. Mature biofilms transition to a low-energy state via the suppression of protein synthesis (*rpl* downregulation) and nutrient transport (*putP* silencing), activating iron acquisition (*bauA, basD*), sulfur salvage (*ssuC*), and nitrogen metabolic pathways to optimize iron-sulfur cluster biogenesis and biofilm stabilization. Pseudotemporal trajectory analysis identified Node 2 as a critical bifurcation point marking the metabolic shift from growth to persistence, a strategy evolutionarily conserved in *Mycobacterium tuberculosis* dormancy [42].

The interrelationships among the identified subpopulations provide insight into biofilm developmental dynamics. The phenylacetate-catabolic Cluster 1 (AB_0 h) and translationally active Cluster 3 (AB_0 h) appear to serve as progenitors for the biofilm-state clusters. Notably, transitional Cluster 0 (M_12 h) retained *paa* gene expression from planktonic Cluster 1 while simultaneously upregulating ribosomal genes characteristic of Cluster 3, suggesting a convergent hybrid state during the planktonic-to-biofilm transition. Pseudotemporal trajectory analysis further corroborates this model: stress-adapted Cluster 7 (M_12 h) and biofilm Cluster 6 (BF_48 h) both committed to biofilm fate (Fate 2) and share functional overlap in NER-mediated DNA repair and ATP-dependent chaperone activity, indicating either lineage continuity or convergent evolution toward long-term stress management. These overlapping features suggest that subpopulations do not undergo abrupt transitions but rather exhibit gradual transcriptional transition with partial retention of ancestral programs, consistent with a continuum model of biofilm maturation.

Several subpopulations identified in this study represent promising therapeutic targets. The iron-acquisition program in Cluster 4 (BF_48 h), centered on *bauA* and *basD*, is a particularly attractive target: gallium-based compounds, which mimic ferric iron and disrupt siderophore-mediated uptake, have demonstrated efficacy against *A. baumannii* biofilm [6]. Recent studies have further demonstrated that the orally bioavailable gallium complex KP46 suppresses planktonic proliferation and disrupts early biofilm development in both susceptible and carbapenem-resistant *A. baumannii* strains [43]. Similarly, the sulfur salvage pathway driven by *ssuC* in Cluster 2 (BF_48 h) and the Tat transport system (*tatC*) in Cluster 11 (BF_48 h) constitute additional vulnerabilities, as disrupting Tat-dependent protein translocation impairs biofilm stability and aminoglycoside tolerance. The nutrient salvage and iron-scavenging programs identified here are particularly relevant to the matrix-encapsulated subpopulations at 48 h. Given that these cells reside within the dense EPS matrix at biofilm maturity, conventional antibiotics face limited penetration. However, combination strategies using matrix-degrading enzymes (e.g., DNase I, proteinase K, or dispersin B) combined with antimicrobial agents have shown enhanced efficacy against mature biofilms in preclinical models [44], suggesting that physical disruption of the matrix may enable access to metabolically embedded subpopulations. Compounds designed to exploit sulfur/iron co-acquisition pathways could potentially reach these cells by hitchhiking on endogenous siderophore or sulfonate transport systems, although whether sufficient drug diffusion through the mature EPS matrix is achievable warrants further experimental validation.

A limitation of this study lies in the temporal resolution of sampling (0 h, 12 h, 48 h), which may obscure transient regulatory events. Additionally, while the transcriptional signatures described here provide a foundation for hypothesis generation, future genetic studies, such as targeted gene knockouts or complementation assays, are required to establish causal relationships between the identified pathways and biofilm fitness. Future work should integrate multi-timepoint (e.g., 6 h, 24 h, 72 h) and multi-omics (metabolome-transcriptome) analyses to resolve the full lifecycle of biofilm dynamics, from nucleation to dispersal, and combine these approaches with functional genetic validation. Such efforts will advance the current precision strategies to combat biofilm-mediated antimicrobial resistance, emphasizing the need for interdisciplinary approaches spanning synthetic biology, microrheology, and targeted drug delivery.

## CRediT authorship contribution statement

**Na Li:** Writing – review & editing, Writing – original draft, Visualization, Validation. **Yue Hu:** Writing – review & editing, Writing – original draft, Visualization. **Lulu Liu:** Writing – review & editing, Writing – original draft, Visualization. **Zhou Chen:** Writing – review & editing, Investigation. **Shuting Huang:** Writing – review & editing, Investigation. **Mingkai Li:** Writing – review & editing, Supervision, Resources, Funding acquisition, Conceptualization. **Xinggang Mao:** Writing – review & editing, Supervision, Funding acquisition, Conceptualization. **Xiaoyan Xue:** Writing – review & editing, Supervision, Resources, Project administration, Funding acquisition, Conceptualization.

## Availability of data and material

The datasets supporting the conclusions of this article are included in the main text and supplementary files. Raw single-cell RNA sequencing data have been deposited in the NCBI SRA database under BioProject accession PRJNA1242596 (https://dataview.ncbi.nlm.nih.gov/object/PRJNA1242596?reviewer=64vmq5dhvtkroc76ekc55dfj14).

## Declaration of competing interests

The authors declare that they have no known competing financial interests or personal relationships that could have appeared to influence the work reported in this paper.

## Data Availability

I have shared the link to my raw data in the relevant section.
