# Supplementary material for: From planktonic to biofilm states: single-cell transcriptomics reveals metabolic reprogramming and cellular heterogeneity in Acinetobacter baumannii
Source: Biofilm. 2026 Aug 21;12:100393. doi: 10.1016/j.bioflm.2026.100393 (PMC13571414; doi:10.1016/j.bioflm.2026.100393)
Supplement: Supplementary file 1 [file mmc1.docx]

**Preparation and detection process of scRNA-Seq samples**

*Acinetobacter baumannii* (*A. baumannii*) was cultured to the logarithmic phase. Planktonic bacteria (AB_0 h) were harvested by centrifugation (1,990 g, 5 min), fixed in 4% paraformaldehyde (PFA) at 4°C for 12 h, washed twice with Phosphate-Buffered Saline with Tween-20 (PBS-T) supplemented with 1 μL of RNase inhibitor, and stored in 20% glycerol. Log-phase bacteria (5 × 10⁶ CFU/mL) were inoculated into 12-well plates (2 mL per well). Mixed planktonic/biofilm (M_12 h) and pure biofilm (BF_48 h) samples were processed identically. Fixed bacteria were pre-incubated at 37°C for 15 min, digested with lysozyme (M20 Genomics, China), washed, and resuspended in PBS containing RNase inhibitor (Thermo Fisher, USA) for single-cell RNA sequencing.

**VITA library preparation and sequencing**

We used the VITAcruizer bacteria preparation instrument DP400 (M20 Genomics, China) to achieve microfluidic droplet generation, single-cell partition and encapsulation, and nucleic acid capture. The VITApilote high-throughput FFPE single-cell transcriptome kit (M20 Genomics, China) was employed for pre-library sample processing, single-cell library construction, and purification. The prepared bacteria suspension was permaeablized and blocked for single-stranded DNA-blocking. Random primers were then added for the reverse transcription of total RNA inside the cell. Subsequently, the generated complementary DNA (cDNA) fragments were ligated to adaptors inside the bacteria. The reverse-transcribed single-bacteria suspension and reagents, along with barcode beads containing cell barcodes and Unique Molecular Identifiers (UMIs), were mixed. The mixture underwent encapsulation, capturing and barcoding using the VITAcruizer DP400 instrument. The resulting product was extended to generate barcoded cDNA strands. Following this, Polymerase Chain Reaction (PCR) amplification was conducted using the cDNA as the template. The purified products from cDNA amplification were used to constructed a standard next-generation sequencing library. The constructed single-cell library contained P5 and P7 adapters and was sequenced on a Xplus sequencer (Illumina) with 150 bp paired-end reads.

**smRandom-seq data processing**

Reads were processed using the VITAseer v1.0 pipeline (M20 Genomics, China) with default and recommended parameters. First, primer sequences and capture adpator sequences were trimmed in raw sequencing data. For each Read1, the UMI (8 nt) and cell-specific barcode (20 nt) were extracted, and sequenced barcodes that could be uniquely assigned to the same accepted barcode (with a Hamming distance ≤ 1 nt) were merged. Read2 was used to generate the gene expression matrix by the STARsolo module in STAR (2.7.10a) [1]. Valid bacteria were identified by STARsolo. The output was then imported into the Seurat (v4.1.1) R toolkit for quality control and downstream analysis of our single-cell RNAseq data.

**Identification of cell types and subtypes by nonlinear dimensional reduction**

After expression matrix filtration, Seurat's default "LogNormalize" method was employed for data standardization. This process involved: (1) normalizing each gene's expression count relative to the total cellular mRNA content, (2) converting values to relative abundance using a scale factor (default: 10,000), and (3) applying natural logarithm transformation [2]. Data from different samples were integrated after identifying ‘anchors’ between datasets using the FindIntegrationAnchors and IntegrateData functions in the Seurat package. The expression of these genes was then scaled, principal component analysis (PCA) was performed on the scaled expression values, and graph-based cluster detection was conducted using the top 30 principal components with the Louvain algorithm for community detection [2]. clusters were visualised in two dimensions with UMAP [3]. For each cluster, the Wilcoxon rank-sum test was used to identify significantly differentially expressed genes compared to the remaining clusters. Cell types were identified using SCINA [4] and known marker genes.

**Differential expression analysis**

Differentially expressed genes (DEGs) between two different samples or clusters were identified using the FindMarkers function in Seurat, based on a likelihood ratio test [5]. DEGs with |log_2_FC| > 0.25 and a *Q value* ≤ 0.05 were considered statistically significant.

**Functional enrichment**

Gene Ontology (GO) enrichment analysis was performed using ClusterProfiler with rigorous statistical controls. Fisher's exact test [6] was used for each GO term based on a 2×2 contingency table constructed from differentially expressed genes in single-cell clusters versus the background gene set. To correct for multiple testing, Bonferroni correction was applied by dividing the significance threshold (α = 0.05) by the total number of GO terms tested [7]. GO terms with adjusted *P values* < 0.05 were considered statistically significant and retained for biological interpretation. GO functional enrichment analysis was carried out using Goatools (https://github.com/tanghaibao/Goatools).

**Pseudotime trajectory analysis**

Pseudotime series analysis was conducted using the R package Monocle2 (v2.22.0) [8]. Briefly, the “differential Gene Test” function in Monocle2 was used to identify DEGs from each cluster. Based on significant DEGs, dimensionality reduction was performed using the DDRTree algorithm [9]. The cell lineage trajectory, based on cell cluster and dimensionality reduction pseudotime, was inferred using the default parameters of Monocle2 after cell ordering, and visualized with the “plot_cell_trajectory” function. Following trajectory construction, DEGs along pseudotime (referred to as “pseudotime-dependent genes”) were identified using the “differentialGeneTest” function. The “plot_genes_in_pseudotime” and “plot_pseudotime_heatmap” functions were used to visualize dynamic changes in pseudotime-dependent gene expression, and significant pseudotime-dependent genes were also displayed in a heatmap. Similarly, the BEAM test in Monocle2 was applied to identify branch-dependent gene expression dynamically expressed along each branch, and significant branch-dependent genes were visualized with a heatmap.

**References**

[1]	Kuijpers, L., Hornung, B., van den Hout-van Vroonhoven, M., van, I.W.F.J., Grosveld, F. and Mulugeta, E., 2024. Split Pool Ligation-based Single-cell Transcriptome sequencing (SPLiT-seq) data processing pipeline comparison. BMC Genomics. 25(1), 361. <https://doi.org/10.1186/s12864-024-10285-3>.

[2]	Stuart, T., Butler, A., Hoffman, P., Hafemeister, C., Papalexi, E., Mauck, W.M., 3rd, et al., 2019. Comprehensive Integration of Single-Cell Data. Cell. 177(7), 1888-1902.e1821. <https://doi.org/10.1016/j.cell.2019.05.031>.

[3]	Leland McInnes, J.H., James Melville, 2020. UMAP: Uniform Manifold Approximation and Projection for Dimension Reduction. arXiv:1802.03426. <https://doi.org/10.48550/arXiv.1802.03426>.

[4]	Zhang, Z., Luo, D., Zhong, X., Choi, J.H., Ma, Y., Wang, S., et al., 2019. SCINA: A Semi-Supervised Subtyping Algorithm of Single Cells and Bulk Samples. Genes (Basel). 10(7). <https://doi.org/10.3390/genes10070531>.

[5]	Kuai, Z. and Hu, Y., 2023. Integration single-cell and bulk RNA-sequencing data to reveal senescence gene expression profiles in heart failure. Heliyon. 9(6), e16214. <https://doi.org/10.1016/j.heliyon.2023.e16214>.

[6]	Choi, L., Blume, J.D. and Dupont, W.D., 2015. Elucidating the foundations of statistical inference with 2 x 2 tables. PLoS One. 10(4), e0121263. <https://doi.org/10.1371/journal.pone.0121263>.

[7]	Klopfenstein, D.V., Zhang, L., Pedersen, B.S., Ramírez, F., Warwick Vesztrocy, A., Naldi, A., et al., 2018. GOATOOLS: A Python library for Gene Ontology analyses. Sci. Rep. 8(1), 10872. <https://doi.org/10.1038/s41598-018-28948-z>.

[8]	Trapnell, C., Cacchiarelli, D., Grimsby, J., Pokharel, P., Li, S., Morse, M., et al., 2014. The dynamics and regulators of cell fate decisions are revealed by pseudotemporal ordering of single cells. Nat. Biotechnol. 32(4), 381-386. <https://doi.org/10.1038/nbt.2859>.

[9]	Qiu, X., Mao, Q., Tang, Y., Wang, L., Chawla, R., Pliner, H.A., et al., 2017. Reversed graph embedding resolves complex single-cell trajectories. Nat. Methods. 14(10), 979-982. <https://doi.org/10.1038/nmeth.4402>.
