# Supplementary material for: From planktonic to biofilm states: single-cell transcriptomics reveals metabolic reprogramming and cellular heterogeneity in Acinetobacter baumannii
Source: Biofilm. 2026 Aug 21;12:100393. doi: 10.1016/j.bioflm.2026.100393 (PMC13571414; doi:10.1016/j.bioflm.2026.100393)
Supplement: Supplementary file 2 [file mmc2.docx]

| Gene | Primers | Primer sequence (5’-3’) | Tm (℃) | Product size (bp) |
| --- | --- | --- | --- | --- |
| *16S rRNA* | Forward | AGCTAACGCGATAAGTAGACCG | 56.51 | 137 |
|  | Reverse | TGTCAAGGCCAGGTAAGGTTC | 57.27 |  |
| *paaA* | Forward | GCTGACCGTGATGACATGATGG | 58.70 | 95 |
|  | Reverse | GCGACATCTGCCCAAGTCAAAG | 59.31 |  |
| *rplB* | Forward | ACCACTTCGTAACATGCCAATCG | 57.79 | 100 |
|  | Reverse | TTGAACAGAAGCACCAGCAGAAC | 57.95 |  |
| *rpsC* | Forward | CAGAAGGTTCATCCAATCGGTATCC | 57.39 | 96 |
|  | Reverse | AAGCAAGTATTCAGCGTATTGTTTCG | 55.42 |  |

**Table S1.** **Sequences of primers used in RT-qPCR**

**Table S2. Summary of the scRNA-seq data.**

| Sample | AB_0 h | M_12 h | BF_48 h |
| --- | --- | --- | --- |
| Estimated Number of Cells | 10,033 | 10,003 | 10,312 |
| Mean Reads per Cell | 499 | 533 | 333 |
| Median Gene per Cell | 158 | 127 | 44 |
| Number of Reads | 251,455,257 | 263,845,680 | 247,157,509 |
| Valid Barcodes | 83.63% | 85.56% | 84.88% |
| Sequencing Saturation | 40.21% | 56.16% | 68.85% |
| Q30_RNA | 96.18% | 95.55% | 95.26% |
| Reads Mapped to Genome | 95.17% | 95.63% | 92.03% |
| Reads Mapped Confidently to Genome | 3.14% | 10.17% | 6.74% |
| Fraction Reads in Cells | 20.89% | 27.91% | 32.74% |
| Total Gene Detected | 3,750 | 3,751 | 3,756 |
| Median UMI per Cell | 229 | 178 | 68 |

Note: Q30_RNA: Read2 sequencing quality value; Reads Mapped to Genome: Proportion of reads mapped to the reference genome, including multi-mapped; Reads Mapped Confidently to Genome: Proportion of reads uniquely mapped to the reference genome.

**Figure S1.** **CLSM images of a 48 h *A. baumannii* biofilm.** FITC‑conA staining (green, extracellular polysaccharides) and Hoechst 33342 staining (blue, bacterial DNA). Scale bar = 10 μm.

**Figure S2. Comparison of subpopulation structures between two independent 48 h biofilm samples.** A) UMAP projection of the two samples (Pre_BF_48 h and BF_48 h), showing overall concordant positions in the reduced dimensional space. B) Clustering results of the two samples. C) Comparison of representative subpopulation distributions between the two samples. D) Correlation matrix between subpopulations of the two samples, with subpopulations containing too few cells excluded from the analysis.

**Figure S3. GO enrichment of subpopulations in BF_48 h sample.** A) GO enrichment of Cluster 8 (ATPbc (ABC)-tc: ATP-binding-cassette (ABC)-transporter-complex). B) GO enrichment of Clusters 10 and 11

**Figure S4. GO enrichment of Cluster 12 and 13**
